# MS Identification of Blood Plasma Proteins Concentrated on a Photocrosslinker-Modified Surface

**DOI:** 10.3390/ijms25010409

**Published:** 2023-12-28

**Authors:** Arina I. Gordeeva, Anastasia A. Valueva, Elizaveta E. Rybakova, Maria O. Ershova, Ivan D. Shumov, Andrey F. Kozlov, Vadim S. Ziborov, Anna S. Kozlova, Victor G. Zgoda, Yuri D. Ivanov, Ekaterina V. Ilgisonis, Olga I. Kiseleva, Elena A. Ponomarenko, Andrey V. Lisitsa, Alexander I. Archakov, Tatyana O. Pleshakova

**Affiliations:** Institute of Biomedical Chemistry (IBMC), 119121 Moscow, Russia; arina.atom@gmail.com (A.I.G.); varuevavarueva@gmail.com (A.A.V.); zx2405@yandex.ru (E.E.R.); motya00121997@mail.ru (M.O.E.); afkozlow@mail.ru (A.F.K.); ziborov.vs@yandex.ru (V.S.Z.); ministreliya13113@gmail.com (A.S.K.); victor.zgoda@gmail.com (V.G.Z.); yurii.ivanov.nata@gmail.com (Y.D.I.); ilgisonisev@gmail.com (E.V.I.); olly.kiseleva@gmail.com (O.I.K.); 2463731@gmail.com (E.A.P.); lisitsa060@gmail.com (A.V.L.); alexander.archakov@ibmc.msk.ru (A.I.A.); topleshakova@yandex.ru (T.O.P.)

**Keywords:** protein immobilization, atomic force microscopy, crosslinker, mass spectrometry

## Abstract

This work demonstrates the use of a modified mica to concentrate proteins, which is required for proteomic profiling of blood plasma by mass spectrometry (MS). The surface of mica substrates, which are routinely used in atomic force microscopy (AFM), was modified with a photocrosslinker to allow “irreversible” binding of proteins via covalent bond formation. This modified substrate was called the AFM chip. This study aimed to determine the role of the surface and crosslinker in the efficient concentration of various types of proteins in plasma over a wide concentration range. The substrate surface was modified with a 4-benzoylbenzoic acid N-succinimidyl ester (SuccBB) photocrosslinker, activated by UV irradiation. AFM chips were incubated with plasma samples from a healthy volunteer at various dilution ratios (10^2^X, 10^4^X, and 10^6^X). Control experiments were performed without UV irradiation to evaluate the contribution of physical protein adsorption to the concentration efficiency. AFM imaging confirmed the presence of protein layers on the chip surface after incubation with the samples. MS analysis of different samples indicated that the proteomic profile of the AFM-visualized layers contained common and unique proteins. In the working series of experiments, 228 proteins were identified on the chip surface for all samples, and 21 proteins were not identified in the control series. In the control series, a total of 220 proteins were identified on the chip surface, seven of which were not found in the working series. In plasma samples at various dilution ratios, a total of 146 proteins were identified without the concentration step, while 17 proteins were not detected in the series using AFM chips. The introduction of a concentration step using AFM chips allowed us to identify more proteins than in plasma samples without this step. We found that AFM chips with a modified surface facilitate the efficient concentration of proteins owing to the adsorption factor and the formation of covalent bonds between the proteins and the chip surface. The results of our study can be applied in the development of highly sensitive analytical systems for determining the complete composition of the plasma proteome.

## 1. Introduction

Blood plasma is the most important sample for proteomic studies. Plasma is a liquid medium with a complex composition and a broad protein concentration range. Plasma is often used in proteomic studies to identify biomarkers for various human pathologies.

Plasma contains major proteins, such as albumin (~60%), globulins (~35%), fibrinogen (~4%), lipoproteins, and proteins involved in iron metabolism (~1%) [1]. A total of 22 proteins account for approximately 99% of all plasma protein content [1]. The remaining 1% of blood protein content is represented by several hundred (or thousand) circulating low-abundant proteins, as well as by proteins secreted by living, apoptotic, and necrotic cells [1]. Proteins present in plasma at all concentration levels are part of the molecular profile of the body, which can be used to assess the health/disease balance. The diversity of plasma proteins can be roughly divided into three classes: proteins present at high concentrations, proteins infiltrated from tissues (“leakage markers”), and cytokines present at low concentrations. “Leakage markers” are extremely important since pathology in a tissue can be detected by measuring their release into the plasma [2]. For instance, plasma levels of cardiac myoglobin (Mb) are 1–85 ng/mL for normal state and 200–1100 ng/mL for myocardial infarction [3]. Meanwhile, cytokines are involved in various infections and disorders affecting the immune system through pro-inflammatory and anti-inflammatory mechanisms [4]. Interleukin (IL)-6 and IL-8 have been shown to predict neonatal sepsis at thresholds of 10.85 pg/mL (sensitivity: 92.5%; specificity: 97.6%) and 60.05 pg/mL (sensitivity: 93.7%; specificity: 65%), respectively [5].

The particular aim of proteomic studies is the identification of proteins that can be potential biomarkers of early-stage disease. The mass spectrometry (MS) method is an indispensable and reliable tool in proteomic research owing to its performance, selectivity, and versatility. To successfully analyze complex biological samples, such as plasma and serum, various functionalized surfaces are often used to concentrate medium- and low-abundance proteins and remove major proteins from the analyzed sample [6]. For instance, the use of immunoaffinity columns allows one to remove up to 20 highly represented proteins and analyze the remaining components by liquid chromatography and tandem mass spectrometry (LC-MS/MS) [7]. The use of the so-called multiple affinity removal system (MARS) was an important step toward improving the detection efficiency of low-abundance proteins. The operation principle of this system is based on the fact that purified antibodies are bound to an inert solid phase and mixed with an antigen solution under conditions favoring adsorption. After antigen capture, unwanted antigens are removed by rinsing, and the purified antigen is released by switching to conditions conducive to desorption [8]. However, this approach can lose a significant amount of information due to the formation of complexes between major proteins and low-abundance proteins of interest [9]. To date, a consensus has not been reached on whether proteins with low plasma concentrations are more “interesting” or clinically relevant than proteins with high concentrations [2]. In addition, the use of affinity columns with immobilized antibodies can be impeded by their high cost, which can be several thousand dollars.

Nanoparticles (NPs) with different physicochemical surface properties are widely employed in plasma proteome profiling. When exposed to biological fluids, a layer of proteins—protein corona—is adsorbed onto the surface of NPs [10]. The protein corona can concentrate serum or plasma components with affinity for the surface [11], which improves the detection of medium- and low-abundance proteins [12]. In their large-scale study, Blum et al. [13] developed an automated protein separation technology platform, Proteograph, which includes a panel of NPs with different physicochemical surface properties, for ex vivo analysis of protein corona formation followed by LC-MS/MS analysis, providing reliable protein detection. These scientists used plasma as a model object and proved the hypothesis that a large panel of NPs can allow the identification of more proteins—particularly those that are present at low concentrations. This approach is predicted to help identify many new biomarkers in the future. In [14], Ma et al. investigated the protein corona formed on zeolite (NaY-PPC) after incubating with plasma to obtain a comprehensive characterization and in-depth profiling of the plasma proteome by LC-MS/MS. They demonstrated that, in the medium, the relative content of low-abundance proteins increased significantly from 2.54% to 54.41%, while that of 20 high-abundance proteins decreased from 83.63% to 25.77%. This method allowed the quantitative identification of approximately 4000 plasma proteins with sensitivity in the pg/mL range—compared with approximately 600 proteins identified in untreated plasma samples. A pilot study, performed with plasma samples obtained from 30 lung adenocarcinoma patients and 15 healthy volunteers, demonstrated that this method can help successfully distinguish between normal and disease states.

Apart from the developed engineered surfaces, including chromatographic columns and nanoparticles described above, the use of atomically smooth functionalized surfaces for concentrating proteins is also promising [15,16]. Atomically smooth substrates are useful for concentrating target proteins in amounts sufficient for their subsequent mass spectrometric identification. In this approach, the atomically smooth substrate with a functionalized surface (i.e., a chip) can be considered an affinity reagent used with a nanotechnology-based detector [17,18]. The latter acts as a quality control device, which allows one to determine the functionalization efficiency of the chip surface and affine complex formation. A striking representative of nanotechnology-based detectors is the atomic force microscope. The use of atomic force microscopy (AFM) in structural biology has made it possible to visualize proteins with high resolution under near-native conditions, as well as to study supramolecular ensembles (protein filaments and viruses) [19]. Thus, the potential of AFM is in the possibility of studying both the “form” of a biological object and its “function”, making it possible to simultaneously solve the problems of proteomics and structural biology [20].

With respect to protein concentration, the use of chemical crosslinking agents (crosslinkers) for modifying atomically smooth surfaces is also promising [16,21,22]. Based on irreversible capturing of the investigated biomolecules from the analyzed solution onto the chemically activated atomically smooth surface of the AFM chip, this approach was called chemical fishing [16]—analogous to molecular fishing [23,24]. A substrate whose surface is partially or fully activated with a crosslinker is called the AFM chip [22]. The use of AFM chips instead of chips with immobilized antibodies or aptamers is reasonable when it is necessary to overcome the thermodynamic limitation of the detection limit. The latter manifests in the dissociation of antibody/antigen complexes formed on the chip surface. Chemical fishing is of use when it is necessary to non-specifically capture all protein molecules from the volume of the analyzed solution to solve protein inventory problems [25].

Previously, optimal conditions were established for the MS identification of bovine serum albumin (BSA) captured from model protein solutions onto the surface of mica chips, modified with either succinimide or benzophenone cross-linker (DSP and SuccBB, respectively) [21]. The use of SuccBB was preferable in the case of MS identification of BSA covalently captured on the chip surface. Upon using the SuccBB crosslinker, the protein on the chip surface was visualized as layers, providing MS identification of more target protein peptides.

In the present study, the applicability of an AFM chip with a SuccBB crosslinker-modified surface to concentrate proteins from a much more complex sample—blood plasma—is demonstrated. Subsequent MS identification of the chip-captured components is performed. The influence of the sample dilution ratio on the number of proteins identified in this sample is also studied.

## 2. Results

### 2.1. AFM Imaging

**Figure A1:**
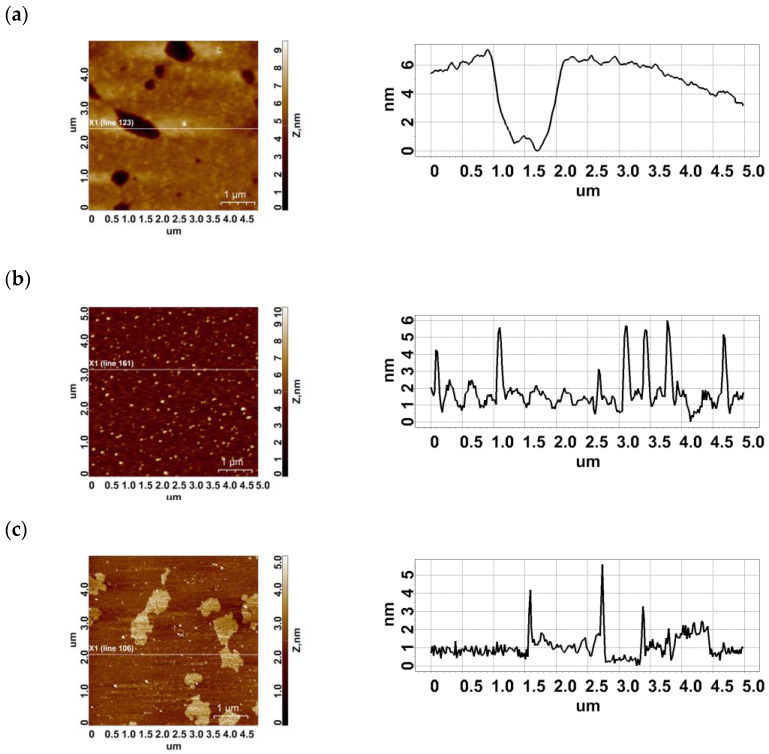
Typical AFM images of the surface of working experiments after incubation of AFM chips with plasma at an appropriate dilution ratio (**left**). (**right**) Cross-section profile corresponding to the lines in the respective AFM images shown on the left: (**a**) #S.1.1UV+, (**b**) #S.1.2UV+, (**c**) #S.1.3UV+.

**Figure A2:**
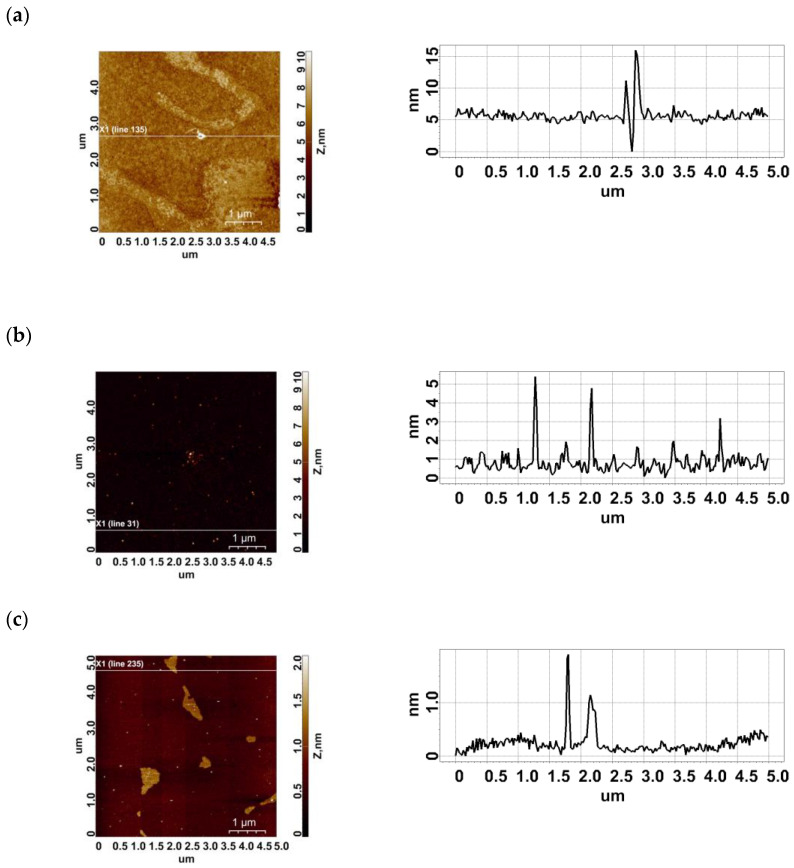
Typical AFM images obtained in experiments on the estimation of physical adsorption after incubation of the AFM chip with plasma at appropriate dilution ratios. Panels on the right display a cross-section profile corresponding to the lines in the respective AFM images shown on the left: (**a**) #S.1.1UV−, (**b**) #S.1.2UV−, (**c**) #S.1.3UV−.

**Figure A3:**
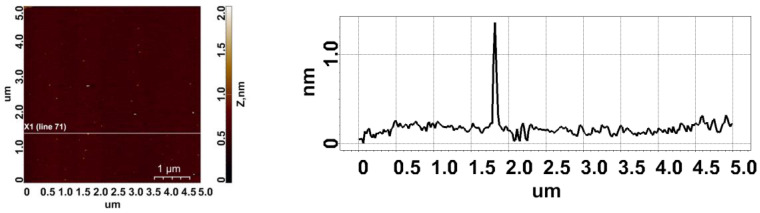
Typical AFM image of the chip surface after incubation in protein-free solution—PBSD buffer (#S.1.4UV+).

The imaging results for the surface of different AFM chips after incubation with plasma samples at various dilution ratios are shown in Appendix A. These results show the AFM data obtained in experimental series 1 (see Section 4.1). After incubation with 10^2^X diluted plasma, layers up to 6 nm in height were visualized on the surface of the #**S.1.1UV+** and #**S.1.1UV−** AFM chips (Figure A1a and Figure A2a, respectively). Following incubation with 10^4^X diluted plasma, compact objects with heights up to 6 nm were visualized on the surface of the #**S.1.2UV+** AFM chip (Figure A1b). In the case of the #**S.1.2UV−** AFM chip, similar objects were observed on its surface, however, there were fewer detected (Figure A2b). After incubation of the #**S.1.3UV+** and #**S.1.3UV−** AFM chips (Figure A1c and Figure A2c, respectively) with samples with the highest dilution (10^4^X), compact objects and layer fragments with heights up to 3 nm were observed on the chip surface. AFM analysis of the control sample #**S.1.4UV+** (Figure A3) revealed that the number of objects with heights > 1 nm did not exceed the noise signal level (500 objects per 400 μm^2^).

The results of AFM imaging indicated that objects visualized in series 1 after incubation of the AFM chips with diluted plasma samples in the working group (UV+) and control group (UV−) could be attributed to protein molecules, as no such objects were visualized on the surface of the control sample (#**S.1.4UV+**)—a solution containing no protein molecules. In most experiments, proteins were adsorbed (control group) or immobilized (working group) in the form of layer fragments, hindering the accurate quantification of proteins on the surface based on the AFM data.

### 2.2. MS Identification of Proteins in Plasma Solution and Eluates from the AFM Chip Surface

**Figure 1:**
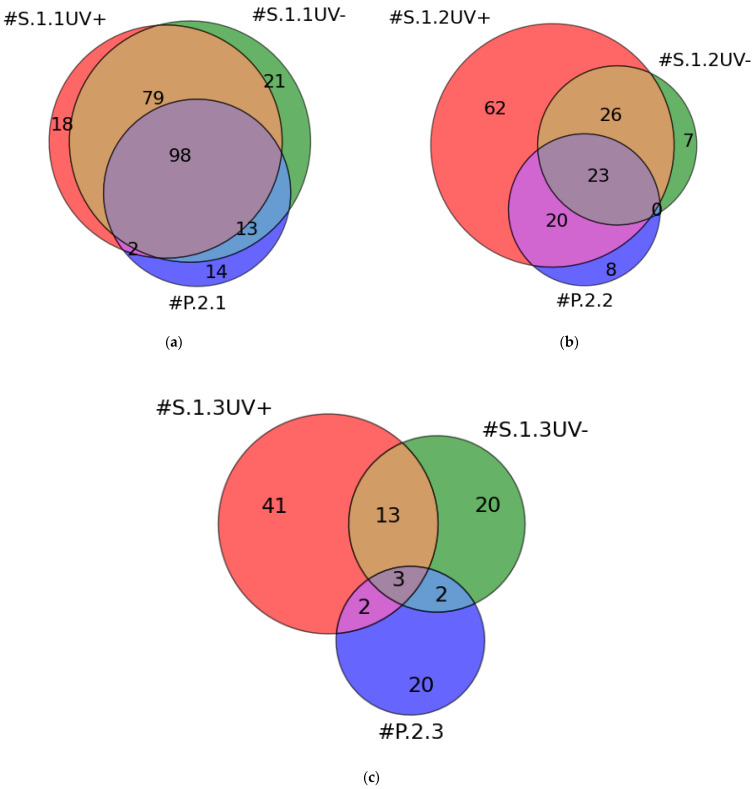
Venn diagrams for MS protein identification. The number of proteins identified on the surface of AFM chips and in plasma samples is presented. Data for dilution ratios of 10^2^X (**a**), 10^4^X (**b**), and 10^6^X (**c**) are shown. Colors indicate the samples from the working group of AFM chips in series 1. Red: chips incubated with plasma samples upon UV irradiation (designated as #S.1.1UV+, #S.1.2UV+, #S.1.3UV+). Green: samples from the control group of AFM chips in series 1—chips incubated with plasma samples without UV irradiation (designated as #S.1.1UV−, #S.1.2UV−, #S.1.3UV−). Blue: plasma samples included in series 2 for which the protein concentration step was omitted (designated as #P.2.1, #P.2.2, #P.2.3).

Figure 1 presents the number of proteins identified by MS in the eluates from the surface of AFM chips incubated with plasma at different dilution ratios (series 1), as well as the number of proteins identified in plasma samples at different dilution ratios (series 2). These results are presented in the form of three-circle Venn diagrams.

As shown in Figure 1a, in the case of protein concentration from plasma with the lowest dilution of 10^2^X, 197 proteins were identified on the surface of the working AFM chip #S.1.1UV+. In the case of the control AFM chip #S.1.1UV−, 211 proteins were identified. At the 10^2^X dilution ratio (#P.2.1), 127 proteins were identified. The number of common proteins characteristic for all three cases was 98. The number of unique proteins identified in each case was 18, 21, and 14, respectively.

As shown in Figure 1b, in the case of protein concentration from plasma at a dilution ratio of 10^4^X, 131 proteins were identified on the surface of the working AFM chip #S.1.2UV+. On the surface of the control AFM chip #S.1.2UV−, 56 proteins were identified. In plasma at the 10^4^X dilution ratio (#P.2.2), 51 proteins were identified. The number of common proteins, characteristic for all three cases, was 23. The number of unique proteins identified in each case was 62, 7, and 8, respectively.

Figure 1c shows that in the case of protein concentration from plasma at the highest dilution ratio of 10^6^X, 59 proteins were identified on the surface of the #S.1.3UV+ working AFM chip. In the case of the control #S.1.3UV− AFM chip, 38 proteins were identified. At the 10^6^X dilution (#P.2.3), 27 proteins were identified in the plasma. The number of common proteins, characteristic for all three cases, was 3. The number of unique proteins identified in each case was 41, 20, and 20, respectively.

As shown in Figure 1, upon increasing the dilution ratio of the analyzed plasma sample, the number of proteins identified on the surface and in the plasma samples decreased as expected. It should be noted that several proteins were only detected on the surface of working AFM chips and control chips. The general trend was an increase in the number of proteins detected on the surface with an increasing dilution ratio. The total number of proteins, largely represented by major proteins, also decreased with an increased dilution ratio.

**Figure 2:**
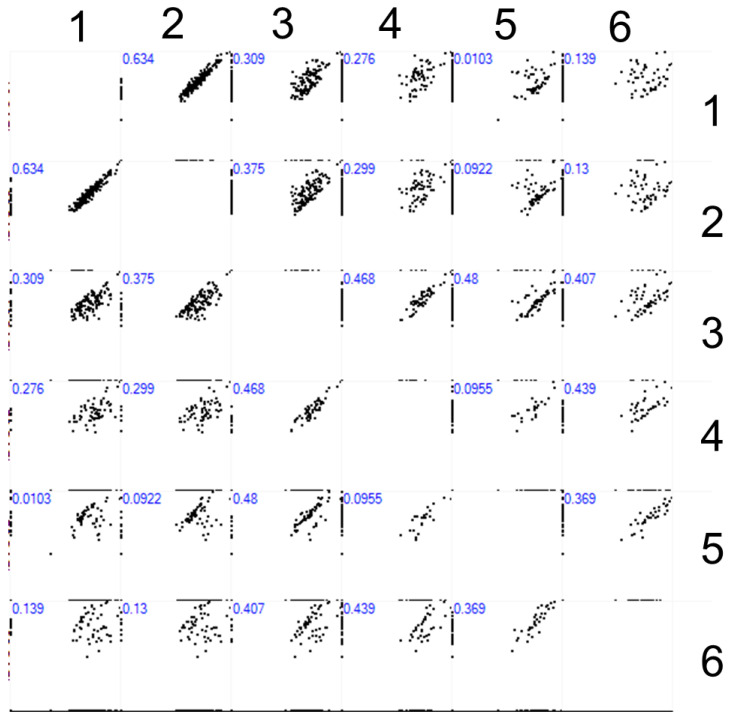
MS identification of proteins concentrated on the surface of AFM chips. Scatter plots are presented as log_2_-transformed intensity-based absolute quantification (IBAQ) values correlation table. Proteins were detected in eluates from the surface after incubation with diluted plasma samples. For each correlation, Pearson’s correlation coefficient is provided in blue. Numbers 1, 3, and 5 correspond to working AFM chips #S.1.1UV+, #S.1.2UV+, and #S.1.3UV+, respectively. Numbers 2, 4, and 6 correspond to control AFM chips #S.1.1UV−, #S.1.2UV−, #S.1.3UV−, respectively. The X and Y axes correspond to the IBAQ values of proteins identified on the respective AFM chips 1–6.

Figure 2 displays the Pearson correlation matrix for analyzing the log_2_-transformed signal intensity obtained for proteins detected on the surface of working and control AFM chips after incubation with plasma samples with (UV+) or without (UV−) UV irradiation. The intensity-based absolute quantification (IBAQ) value represents the sum of all peptide peak intensities divided by the number of theoretically observable tryptic peptides [26].

Figure 2 presents the comparison of MS-identified proteins on the surface of working and control AFM chips. For #S.1.1UV+ and #S.1.1UV− chips, after incubation with plasma at a dilution ratio of 10^2^X, a considerable correlation (Pearson’s coefficient = 0.634) was observed. By increasing the dilution ratio to 10^4^X, the correlation ratio between #S.1.2UV+ and #S.1.2UV− samples decreased to moderate (Pearson’s coefficient = 0.468). At the highest dilution ratio (10^6^X), the correlation between samples #S.1.3UV+ and #S.1.3UV− remained moderate, while the Pearson coefficient decreased to 0.369.

Analysis of the results obtained for working AFM chips incubated with plasma samples at various dilution ratios upon UV irradiation revealed a poor correlation between samples #S.1.1UV+ and #S.1.2UV+ (Pearson’s coefficient = 0.309). Comparing the #S.1.1UV+ and #S.1.3UV+ samples, virtually no correlation was observed (Pearson coefficient = 0.0103). Comparing samples #S.1.2UV+ and #S.1.3UV+, a moderate correlation with a Pearson’s coefficient of 0.48 was obtained. Thus, the composition of proteins captured on the surface of the working AFM chip after incubation with plasma at the highest dilution ratio (#S.1.3UV+) differed significantly from that of proteins concentrated from the plasma at the lowest dilution ratio (#S.1.1UV+).

**Figure 3:**
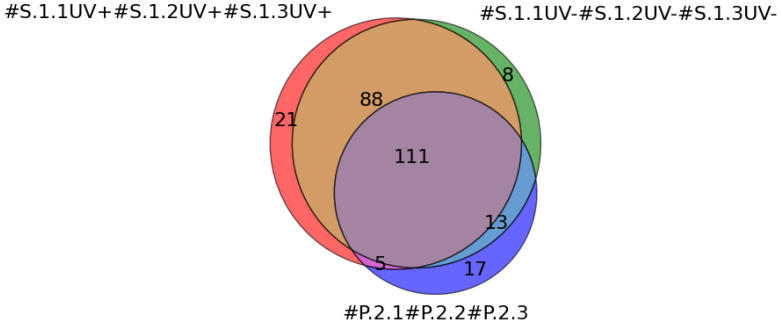
Venn diagram summarizing the total number of all proteins identified on the surface of the working AFM chips after incubation with plasma samples. Colors indicate the samples from the working group of AFM chips in series_1—chips incubated with plasma samples under irradiation (signatures #S.1.1UV+, #S.1.2UV+, #S.1.3UV+; red); samples from the control group of AFM chips in series_1—chips incubated with plasma samples without irradiation (signatures #S.1.1UV−, #S.1.2UV−, #S.1.3UV−; green); and plasma samples included in series_2, in which no surface protein concentration step was performed (signatures #P.2.1, #P.2.2, #P.2.3; blue).

Figure 3 displays a three-circle Venn diagram summarizing the total number of all proteins identified on the surface of the working AFM chips (#S.1.1UV+, #S.1.2UV+, #S.1.3UV+, red), control AFM chips (#S.1.1UV−, #S.1.2UV−, #S.1.2UV−, #S.1.3UV, green), and plasma with different dilution ratios (#P.2.1, #P.2.2, #P.2.3, blue).

As can be seen from Figure 3, a total of 225 proteins were identified on the surface of working AFM chips (UV+) after incubation with plasma at various dilution ratios, with 21 unique proteins differing from other cases. For the case of control AFM chips (UV−), 220 proteins were identified, with 8 unique proteins. In plasma, a total of 146 proteins were detected, with 17 unique proteins. These data indicate that the use of AFM chips allows the identification of more proteins with a significant presence of unique proteins after incubation with diluted plasma samples.

Table 1 lists the proteins identified on the surface of the working AFM chips (#S.1.1UV+, #S.1.2UV+, #S.1.3UV+).

As can be seen from Table 1, most unique proteins (12) were identified on the surface of the working AFM chips after incubation with plasma at the lowest dilution ratio (#**S.1.1UV+**). With an increase in the dilution ratio (#**S.1.2UV+**), 2 more unique proteins—Centrosomal and Histidine ammonia-lyase—were identified. At the highest dilution ratio (#**S.1.3UV+**), 3 unique proteins (Corneodesmosin, Fatty acid-binding protein 5 and A-kinase anchor protein 9) were identified. Glyoxylate reductase/hydroxypyruvate reductase was detected on the surfaces of all working AFM chips.

**Figure 4:**
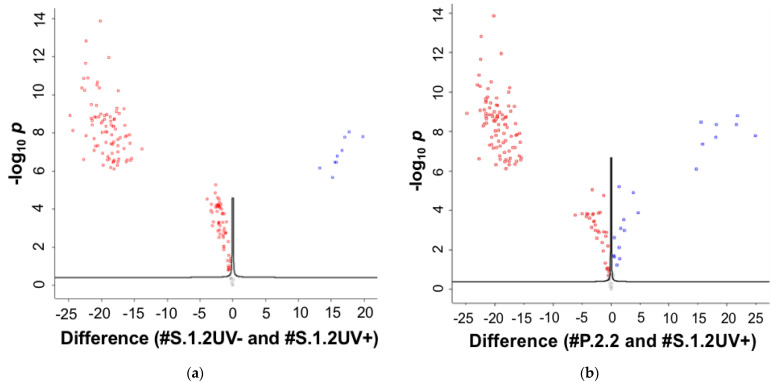
Volcano plots displaying the MS identification of proteins by plasma analysis at a dilution ratio of 10^4^X with and without the concentration step. (**a**) Comparison of samples with #S.1.2UV+ (red dots) and #S.1.2UV− (blue dots), (**b**) Samples with #S.1.2UV+ (red dots) and #P.2.2 (blue dots) are presented. The X-axis shows the log_2_ of the fold-change value (log_2_(B/A)) of differential protein expression across sample groups, and the Y-axis shows the negative log_10_ of the *p* value reflecting changes in gene expression. Each dot in the figure represents a protein. Comparisons between different protein groups are indicated by different colors.

Focusing on the independent changes in protein levels, univariate statistical analysis was employed to reliably detect the significant differences in protein expression comparing #**S.1.2UV+** with #**S**.**1.2UV−** and then only with plasma **#P.2.2**. Volcano plots for the binary comparisons are shown in Figure 4, representing a scatter plot displaying the dependence of statistical relevance (*p* value) on fold-change. That is, the Volcano plot shows the negative log_10_ of the *p* value (−log_10_*p*; Y-axis) plotted against the log_2_ of the fold-change values between the groups (X-axis).

## 3. Discussion

In the present work, the applicability of an AFM chip with a SuccBB crosslinker-modified surface was tested on the concentration of proteins from blood plasma, followed by MS identification of the chip-captured proteins. The original plasma sample was divided into aliquots and used for analysis at different dilution ratios (10^2^X, 10^4^X, 10^6^X) to estimate the effect of the concentration factor on the efficiency of capturing a particular type of protein on the chip surface.

It has been shown that the total number of proteins detected with and without applying the concentration step decreased with increasing dilution ratio. This decrease was expected since the concentration of all proteins decreases when plasma is diluted. However, it should be noted that the composition of the protein mixture changes with increasing dilution. Dilution of plasma with buffer possibly affects the physical properties of proteins and protein–protein complexes. This can lead to changes in the spatial conformation of proteins or affect the strength of the intermolecular interactions. Namekar et al. [27] reported that serum dilution can serve as a critical parameter that can affect the results of Luminex-based microsphere immunoassays (MIA) upon detection of antibodies against West Nile Virus (WNV). Serum dilution helps eliminate the influence of complement proteins or the prozone effect observed in the case of high antibody titers and thus may improve the sensitivity of MIA [28]. Meanwhile, increased serum dilution can decrease sensitivity [29]. MIA of WNV was performed at a serum dilution of 1:100 [30], whereas other MIAs used a lower serum dilution (1:20) as this resulted in low levels of nonspecific background signals within a high dynamic range of signal intensities [31].

Upon using a concentration step, the number of proteins identified on the surface of working AFM chips tends to increase in the case of diluted plasma. The working chips have been used under conditions that allow the covalent binding of proteins to the surface (see Figure 1). However, before the covalent binding reaction between the protein groups and the crosslinker’s active group on the chip surface, protein adsorption on this surface must occur [32]. Protein adsorption on the solid substrate surface can occur at the expense of electrostatic and hydrophobic interactions [33,34] by forming hydrogen bonds [34] or van der Waals forces [35,36]. Since plasma proteins differ in molecular weight, structure, and concentration, they may interact with the surface in different ways [34]. This leads to differences in the composition of the protein layer, as indicated by the results obtained for working and control AFM chips. In the case of the control AFM chips, their surface is identical to that of working AFM chips, however, the photocrosslinker activation is not performed, i.e., there is no UV irradiation. The benzophenone molecule (active group of SuccBB) is characterized by significant hydrophobicity [37]. The presence of SuccBB on the chip surface may contribute to partial denaturation of proteins and thus lead to efficient adsorption and further concentration of proteins [38]. This may explain the presence of a significant number of proteins (211) in the case of the lowest dilution ratio. Thus, 197 proteins were identified on the surface of the control AFM chip #S.1.1UV−, and 197 were identified on the working AFM chip #S.1.1UV+. With an increase in the plasma dilution ratio (i.e., decreasing the amount of protein), the contribution of covalent binding between the protein and the SuccBB-modified surface presumably increased. Thus, 131 proteins were identified on the surface of the #S.1.2UV+ AFM chip; 62 proteins were not detected in the case of the #S.1.2UV− AFM chip or in the plasma sample at a dilution ratio of 10^4^X (#P.2.2). The carbon–hydrogen bond (C–H bond) of a protein molecule must be within 3.1 Å of the carbonyl oxygen of benzophenone to allow a covalent crosslinking reaction between the protein and surface [37]. At an increased plasma dilution ratio and upon agitation, proteins can approach the surface within the distance required for covalent binding. In addition, a decrease in protein concentration with plasma dilution results in less UV absorption/scattering necessary for the activation of SuccBB on the surface.

As shown in the three-circle Venn diagram displaying the total number of proteins (Figure 3), 21 unique proteins were detected on the surface of the working AFM chips (UV+). Most unique proteins were identified on the surface of the **#S.1.1UV+ AFM** chip (12 proteins) after incubation with the least diluted plasma sample. However, additional proteins were identified by increasing the dilution ratio. Thus, in the case of the **#S.1.2UV+** AFM-chip, two additional unique proteins—Centrosomal and Histidine ammonia-lyase—were identified. In the case of the AFM-chip **#S.1.3UV+,** three more unique proteins—Corneodesmosin, Fatty acid-binding protein 5 and A-kinase anchor protein 9—were additionally identified (Table 1). Meanwhile, glyoxylate reductase/hydroxypyruvate reductase was identified on the surface of all working AFM chips. It is worth noting that these proteins do not pertain to major plasma proteins. For instance, Plakophilin-1 and Calmodulin-like protein 5 were identified on the surface of AFM-chips **#S.1.2UV+** and **#S.1.3UV+** in both cases. Titin protein was found on the surface of the **#S.1.1UV+** and **#S.1.3UV+** AFM chips in both cases.

It should be noted that upon studying plasma proteins, the maximum number of proteins that can be captured onto the AFM chip (i.e., the capacity of the AFM chip) can represent a limitation of the AFM-MS approach proposed herein. The limited capacity of the AFM chip hinders the capturing of more proteins necessary for MS identification. However, this limitation can be overcome by using several chips to analyze the same sample. Meanwhile, our AFM data indicate that the limited capacity of the AFM chip leads to multilayer adsorption of protein. That is, while the first protein layer is covalently bound to the chip surface, subsequent protein layers can physically adsorb onto this first layer. In their review [39], Brash et al. reported that an increase in the protein layer thickness upon adsorption can be explained by binding complement proteins with the proteins that had already been adsorbed. Arvidsson et al. [40] demonstrated that silicon surface adsorbs a 3–5-nm-thick protein layer in one minute.

Solving the problem of controlled adsorption of these protein layers is required for further successful use of our proposed approach.

Furthermore, the established list of unique proteins for the working series of experiments will likely differ in the analysis of diluted blood plasma from other volunteers. To confirm this hypothesis, further investigation involving a larger number of samples and a special design of the ongoing experiment is required.

## 4. Materials and Methods

### 4.1. Experiment Setup

**Figure 5:**
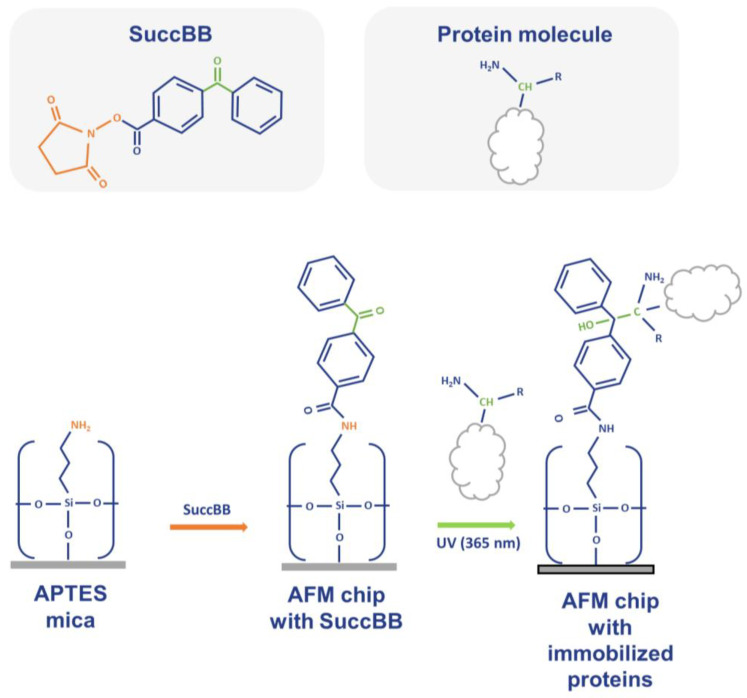
Schematic representation of the processes occurring on the AFM chip surface. Orange letters highlight the functional groups participating in the formation of chemical bonds upon the modification of the substrate surface with the SuccBB crosslinker (orange arrow). Green letters highlight the functional groups participating in forming covalent bonds between the crosslinker-modified AFM chip surface and captured protein upon UV irradiation during the incubation of the chip in the analyzed sample (green arrow).

Figure 5 schematically illustrates chemical reactions that occur upon modification of the substrate surface with the SuccBB crosslinker and during incubation of the AFM chip with diluted protein-containing plasma samples.

**Figure 6:**
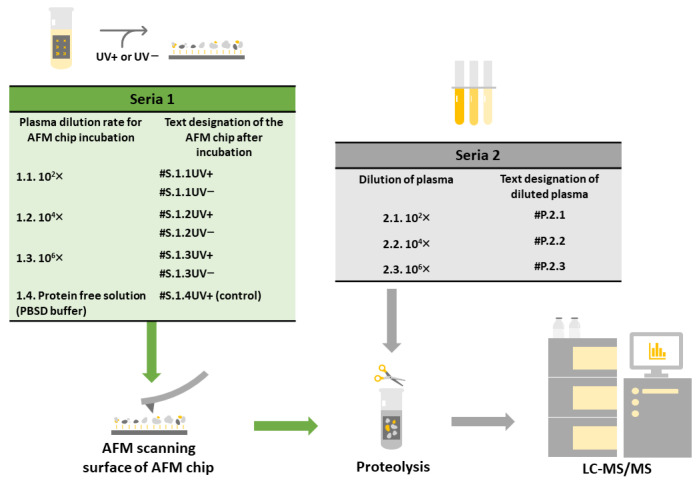
Schematic of the experiment workflow. The main steps of the experiment are described in the text. The left panel shows the steps related to the work using AFM chips, while the right panel shows the work with plasma samples without concentration on the chip surface.

Figure 6 presents a schematic of an experiment comprising two series. In series 1, proteins concentrated on the surface of the AFM chip were identified (left panel in Figure 6).

In this series, AFM chips with their surface modified with the SuccBB crosslinker were incubated in blood plasma at the respective dilution ratios: 10^2^X, 10^4^X, or 10^6^X. In the working group, AFM chips were incubated in plasma under UV irradiation. Labeling of samples in this group #**S.1.1 UV+**, #**S.1.2 UV+**, #**S.1.3 UV+** was performed according to the dilution ratio. To estimate the contribution of adsorbed (not covalently attached) proteins on the surface, in a series of experiments in the control group, AFM chips were incubated with plasma in the absence of UV (#**S.1.1 UV−**, #**S.1.2 UV−,** #**S.1.3 UV−**). In a separate blank experiment to estimate the influence of possible external contamination, the SuccBB-modified AFM chip was incubated under UV irradiation in protein-free PBSD buffer to dilute the plasma (#**S.1.4 UV+**). After incubation, all chip surfaces were washed and subjected to AFM analysis for surface visualization, followed by a sample preparation (proteolysis) step for MS analysis. Trypsinolysis was performed on the chip surface to cleave the proteins. Then, the peptide mixture was eluted from the surface, and the eluates were transferred to the LC-MS/MS stage for protein identification.

In series 2, protein identification was performed in plasma samples at various dilution ratios of 10^2^X, 10^4^X, and 10^6^X (right panel in Figure 6). The samples in this series were labeled #P.2.1, #P.2.2, and #P.2.3. Plasma samples after the sample preparation step (trypsinolysis) were submitted for further identification by liquid chromatography with tandem mass spectrometry (LC-MS/MS).

It should be emphasized that the plasma aliquots used in series 1 and 2 were obtained using the same biological sample from the same healthy volunteer.

### 4.2. Proteins

Porcine trypsin was from Promega Corp. (Cat.# V5111, Madison, WI, USA). Promega Sequencing Grade Modified Trypsin is a porcine trypsin modified by reductive methylation, rendering it resistant to proteolytic digestion.

### 4.3. Plasma Sample Preparation

In the study, a plasma sample of a conditionally healthy volunteer was analyzed. For AFM analysis, 10 μL of plasma was added to 990 μL of Dulbecco’s modified phosphate buffered saline (PBSD), corresponding to a dilution of 10^2^X of the original sample. Dilutions of 10^4^X and 10^6^X were prepared by serial hundred-fold dilutions. Briefly, the initial plasma sample was divided into aliquots, which were further used for analysis at various dilution ratios (10^2^X; 10^4^X; 10^6^X). Various dilution ratios were tested to estimate the influence of the concentration factor on the efficiency of adsorption of a particular type of protein onto the surface. For instance, the molar concentration of the major protein albumin in plasma is approximately 6.4 × 10^−4^ mol/L [41]. Thus, at a dilution of 10^2^X, the albumin concentration will be 6.4 × 10^−6^ mol/L, at 10^4^X, it will be 6.4 × 10^−8^ mol/L, and at 10^6^X, it will be 6.4 × 10^−10^ mol/L. The 10^2^X dilution of plasma was selected based on data reported previously in studies on biospecific molecular fishing: the use of plasma at such a dilution allows one to avoid nonspecific adsorption of biological macromolecules on the surface of the AFM chip with immobilized molecular probes [17]. The maximum dilution ratio tested in this study was 10^6^X, at which the final albumin concentration was close to the detection limit attainable by commonly used experimental proteomic methods (10^−12^ mol/L). A dilution ratio of 10^4^X was used as an intermediate point. Different dilution ratios result in a shift of the dynamic range and in lower concentrations of major plasma proteins, which can influence the efficiency of capturing medium- and low-abundance proteins onto the AFM chip.

This research was approved by independent ethical committees from the organizations that provided the samples. Written informed consent was obtained from all healthy volunteers for participation in the study and the use of biological material.

### 4.4. Chemicals

The following reagents were used in the study: 4-benzoylbenzoic acid N-succinimidyl ester (SuccBB) (Sigma, St. Louis, MO, USA), 3-aminopropyltriethoxysilane (APTES; Acros Organics, Fair Lawn, NJ, USA), dimethyl sulfoxide (DMSO; Sigma, St. Louis, MO, USA), Emulgen 913 (Kao Atlas, Osaka, Japan), triethylammonium bicarbonate (TEAB) buffer (1 M, for HPLC; Honeywell Fluka, Washington, DC, USA), 100% acetonitrile for HPLC (ACN; Merck, Darmstadt, Germany), isopropanol, and formic acid (ACROS, Morris Plains, NJ, USA).

PBSD buffer was prepared by dissolving a salt mixture, commercially available from Pierce, in ultrapure water. All solutions used in this study were prepared using deionized ultrapure water (resistivity, 18.2 MΩ × cm) obtained with a Simplicity UV system (Millipore, Molsheim, France).

### 4.5. Modification and Activation of AFM Substrate Surface

Muscovite mica sheets (SPI, West Chester, PA, USA) were cut into 7 × 15 mm pieces and used to prepare the AFM substrates. The surface of muscovite mica substrates was modified with (3-aminopropyl)triethoxysilane (APTES) using a vapor-phase deposition technique developed by Yamada et al. [42].

A 0.31 mM SuccBB solution was obtained by dissolving a weighed quantity of SuccBB in DMSO (final volume of the tube containing 1 mL of the SuccBB solution and incubated at room temperature with the activation solution was 1 mL). Next, the AFM substrate was immersed in an Eppendorf test tube containing the activation solution. The tube was placed into a shaker and incubated therein at 120 rpm for 18 h. After incubation, the AFM substrate was washed once with 1 mL of DMSO: EtOH (1:1) at 40 °C for 30 min and then twice with 1 mL of 50% (*v*/*v*) EtOH for 30 min. After the washing, the AFM chip was dried in a nitrogen stream and used for protein immobilization.

### 4.6. Fishing the Plasma Proteins

In the case of a SuccBB-activated surface, the AFM substrate was incubated with 1 mL of volunteer plasma of varying dilution in 10^2^X, 10^4^X, and 10^6^X in a rotating test tube at 200 rpm under UV or without UV irradiation for 1 h. The irradiation was performed using a UVP Crosslinker CL-3000L device (Analytik Jena US, Upland, CA, USA) at a wavelength of 365 nm. The distance between the test tube and the UV light source was ~10 cm. The designation of AFM chips incubated in plasma at appropriate dilution ratios under UV irradiation was #**S.1.1UV+**, #**S.1.2UV+**, #**S.1.3UV+** (series 1, working group). The designation of AFM-chips incubated in plasma at an appropriate dilution ratio in the absence of UV: #**S.1.1UV−**, #**S.1.2UV−**, #**S.1.3UV−** (series 1, control group—adsorbed but not covalently crosslinked protein molecules on the chip surface).

AFM substrates activated with SuccBB and immobilized with the protein as described above were washed once with 1 mL of 0.01% aqueous solution of Emulgen 913 at 37 °C for 30 min and twice with 1 mL of ultrapure water at 37 °C for 30 min; they were then dried in air.

Control experiments were performed to estimate the amount of non-protein particles adsorbed on the surface; crosslinker-activated AFM substrates were incubated in a protein-free solution PBSD under UV irradiation (#**S.1.4UV+**).

### 4.7. AFM Scanning

In control and working experiments, the AFM chip surface was scanned in the tapping mode using Titanium and Solver NexT atomic force microscopes (NT-MDT, Zelenograd, Russia). NSG10 cantilevers (TipsNano, Zelenograd, Russia) with a tip curvature radius of 6–10 nm, a resonance frequency of 47–150 kHz, and a force constant of 0.35–6.1 N/m were used. The scan size was 5 × 5 µm (resolution, 256 × 256 points); at least 10 scans of different substrate areas were acquired for each substrate. A TGZ1 grating (Zelenograd, Russia) with a step height of 21.4 ± 1.5 nm was used to calibrate the microscope.

Processing of AFM images was performed using standard NovaPx 3.5.0 software and involved subtraction of the 2nd order plane. The height of AFM-detected objects was the main criterion for determining their size [22].

### 4.8. Preparation of the AFM Substrate for Mass Spectrometry Measurements

For trypsinolysis, the chips were immersed in a solution containing 10 µL of trypsin (0.18 g/L), 20 µL of TEAB (50 mM), and 600 µL of deionized water so that the solution completely covered the mica. Trypsinolysis was performed on an incubator shaker at 37 °C and 140 rpm for 18 h and then in a heat chamber at 40 °C for 2 h in a horizontal position to cover the mica with the buffer. Next, the chips immersed in a tryptic mixture were placed into a shaker at 40 °C for 60 min. Then, the samples were shaken with a SkyLine vortex (Elmi Ltd., Rīga, Latvia) in MIX1 mode and centrifuged at 9000 rpm for 10 min. The hydrolysis was stopped with 100 μL of 0.1% formic acid. The solutions were prepared in the same way as chips.

After trypsinolysis, mixtures of peptides were dried in a vacuum centrifuge SpeedVac (Eppendorf, Macquarie Park, NSW, Australia). Then, the dry mixtures were prepared for LC-MS/MS analysis by first dissolving them in 5% (*v*/*v*) formic acid.

### 4.9. MS Measurements

#### 4.9.1. LC-MS Measurements

Panoramic mass spectrometric analysis was carried out on a sensitive Q Exactive HF mass spectrometer (HF Hybrid Quadrupole Orbitrap mass spectrometer, Thermo Fisher Scientific™, Rockwell, IL, USA), which allows panoramic protein search and is used in routine analysis.

The peptides for each sample were separated by high-performance liquid chromatography (HPLC, Ultimate 3000 Nano LC System, Thermo Scientific, Rockwell, IL, USA) on a 15 cm long 75 µm id C18 column (Acclaim^®^ pepmap™ RSLC, Thermo Fisher Scientific, Rockwell, IL, USA).

The peptides were eluted with a gradient from 5–35% buffer B (80% acetonitrile, 0.1% formic acid) over 115 min at a flow rate of 0.3 µL^−1^ min. The total run time was 90 min to reach 99% buffer B, 10 min to wash with 99% buffer B, and 15 min to re-equilibrate with buffer A (0.1% formic acid).

#### 4.9.2. Acquisition of MS Data

Mass spectrometric measurements were carried out according to the procedure described in a previous study [43]. The analysis was performed using a Q Exactive HF mass spectrometer (HF Hybrid Quadrupole Orbitrap mass spectrometer, Thermo Fisher Scientific™, Rockwell, IL, USA). Mass spectra were obtained with a resolution of 60,000 (MS) and 15,000 (MS/MS) in the m/z range 400–1500 (MS) and 200–2000 (MS/MS).

The resulting RAW files, unprocessed with a mass spectrometer, were analyzed with the maxquant program (version 1.5.5.1, Jurgen Cox, Max Planck Institute for Biochemistry, Martinsried, Germany) [44] with the built-in Andromeda search system [45].

Protein N-terminal acetylation and methionine oxidation were variable modifications for the peptide search. A maximum mass deviation of 5 ppm was allowed for precursor identification, and 20 ppm was set as a tolerance for fragment identification. For trypsin digestion, 2 missing sites were allowed. The false discovery rate (FDR) of resulting protein identifications was 0.01.

#### 4.9.3. MS Data Processing

To perform molecular profiling of the proteomic composition of biological samples caught on the AFM chip, panoramic analysis was performed using a Q Exactive HF mass spectrometer (Thermo Scientific, Waltham, MA, USA). Data from mass spectrometers were processed using bioinformatics. For panoramic analysis performed using a Q Exactive HF mass spectrometer, the results were processed in MaxQuant software version 2.2.0.0.

The results were classified into two peptides, and the more contaminated proteins were removed from the outside, as well as the reverse protein. For statistical processing, the control (PBSD mica) was subtracted from the experiments. For data visualization, the IBAQ parameter [26] was used. As was noted in Section 2.2, the IBAQ value represents the sum of all peptide peak intensities divided by the number of theoretically observable tryptic peptides [26]. According to international standards of reliable identification, proteins were considered to be detected if at least two unique peptides were present [46].

## 5. Conclusions

The results reported indicate that AFM chips, whose surface is modified with a SuccBB crosslinker, allow one to efficiently perform protein concentration from the liquid sample owing to the adsorption factor and the possibility of covalent bonding between proteins and the chip surface. The contribution of the adsorption factor is higher in the case of analysis of plasma samples at a lower dilution ratio. We believe that the AFM chips developed herein can be applied to solve various biomedicine problems. For instance, they can be employed for the targeted search of antigens upon immobilization of antibodies or aptamers via crosslinkers onto the surface of an AFM chip and the nonspecific capturing of components from a biological sample or their depletion. The proposed approach can also help study the blood plasma proteome to determine its complete composition and identify proteins, allowing one to distinguish between normal and pathological states.

## Figures and Tables

**Table 1:** Unique proteins identified on the surface of working AFM chips #S.1.1UV+, #S.1.2UV+, #S.1.3UV+.

#S.1.1UV+	#S.1.2UV+	#S.1.3UV+
Fructose-1,6-bisphosphatase 1	Centrosomal protein of 290 kDa	Plakophilin-1
Myosin regulatory light chain 12A	Histidine ammonia-lyase	Corneodesmosin
Flavin reductase	Plakophilin-1	Titin
Transgelin-2	Calmodulin-like protein 5	A-kinase anchor protein 9
Ras-related protein Rab-7a	Glyoxylate reductase/hydroxypyruvate reductase	Calmodulin-like protein 5
Mesencephalic astrocyte-derived neurotrophic factor		Glyoxylate reductase/hydroxypyruvate reductase
Thymosin beta-4		Fatty acid-binding protein 5
Septin-7		
Protein DBF4 homolog B		
Titin		
Glyoxylate reductase/hydroxypyruvate reductase		
Myosin-2		
Coronin-1C		
Polymeric immunoglobulin receptor		

Proteins identified on all three chips are highlighted in green. Proteins identified on #S.1.1UV+ and #S.1.3UV+ chips are highlighted in blue. Proteins identified on #S.1.2UV+ and #S.1.3UV+ chips are highlighted in yellow.

## Data Availability

The data underlying the study can be obtained from the corresponding author upon reasonable request.

## Appendix A

Figure A1 shows typical AFM images of the surface of working experiments after incubation with plasma at the corresponding dilution ratio: **#S.1.1UV+**, **#S.1.2UV+**, **#S.1.3UV+**. The white line indicates the cross-section.

Figure A2 shows typical AFM images of the surface to evaluate physical adsorption after incubation with plasma of the corresponding dilution ratio: #**S.1.1UV−**, #**S.1.2UV−**, and #**S.1.3UV−**. The white line indicates the cross-section.

Figure A3 shows AFM images of the surface after incubation in protein-free solution (PBSD buffer): #**S.1.4UV+**. The white line marks the cross-section.
